# A Case of Rapidly Proliferative Glomerulonephritis Secondary to Syphilis That Responded to Treatment With Penicillin

**DOI:** 10.7759/cureus.13468

**Published:** 2021-02-21

**Authors:** Raheel S Siddiqui, Vikram Sumbly, Adriana Abrudescu

**Affiliations:** 1 Internal Medicine, Icahn School of Medicine at Mount Sinai (New York City Health and Hospitals/Queens), Jamaica, USA; 2 Rheumatology, Icahn School of Medicine at Mount Sinai (New York City Health and Hospitals/Queens), Jamaica, USA

**Keywords:** rapidly proliferative glomerulonephritis, syphilis, crescentic glomerulonephritis, kidney involvement in syphilis

## Abstract

The involvement of kidneys in syphilis has been reported in the literature with the majority of cases presenting with nephrotic-range proteinuria. We report a case of rapidly proliferative glomerulonephritis in a patient with secondary syphilis. A 40-year-old male with a history of human immunodeficiency virus (HIV), chronic hepatitis B virus, and chronic kidney disease stage 2 presented with fatigue, anorexia, weight loss, arthralgia, chills, and rash throughout the body. The patient was non-compliant with HIV medication and had unprotected sexual intercourse. Labs showed blood urea nitrogen of 57 mg/dL (range: 7-23 mg/dL), creatinine 8.2 mg/dL (range: 0.5-1.3 mg/dL), and high titers of rapid plasma reagin. The biopsy showed crescentic glomerulonephritis with c3 deposition in mesangium and basement membrane. The patient responded to treatment with penicillin therapy with gradual improvement in kidney function.

## Introduction

Syphilis is a bacterial infection caused by the spirochete *Treponema pallidum*. The majority of the cases are transmitted by sexual contact, but it can also be transmitted congenitally during transmission through the birth canal [1]. The usual clinical course involves progression through different stages such as the incubation period, primary stage, secondary stage, and late or tertiary stage. Although the primary stage involves only painless chancre and regional lymphadenopathy, the secondary stage is characterized by a wide variety of systemic involvement such as periostitis, hepatitis, glomerulonephritis, uveitis, and meningitis in addition to skin manifestations [2]. In the United States, the incidence of primary and secondary syphilis was at an all-time low with 2.1 cases per 100,000 persons in 2000; since then, incidence has been increasing, particularly in men who have sex with men, with a 15.2% increase between 2006 and 2007 [3].

The involvement of kidneys in syphilis has been reported in the literature with the majority of the cases presenting with nephrotic-range proteinuria [4]. Rapidly proliferative glomerulonephritis is rarely reported in syphilis patients [5,6]. We report a case of rapidly proliferative glomerulonephritis in a human immunodeficiency virus (HIV) patient with secondary syphilis that was successfully treated with penicillin.

## Case presentation

**Figure 1:**
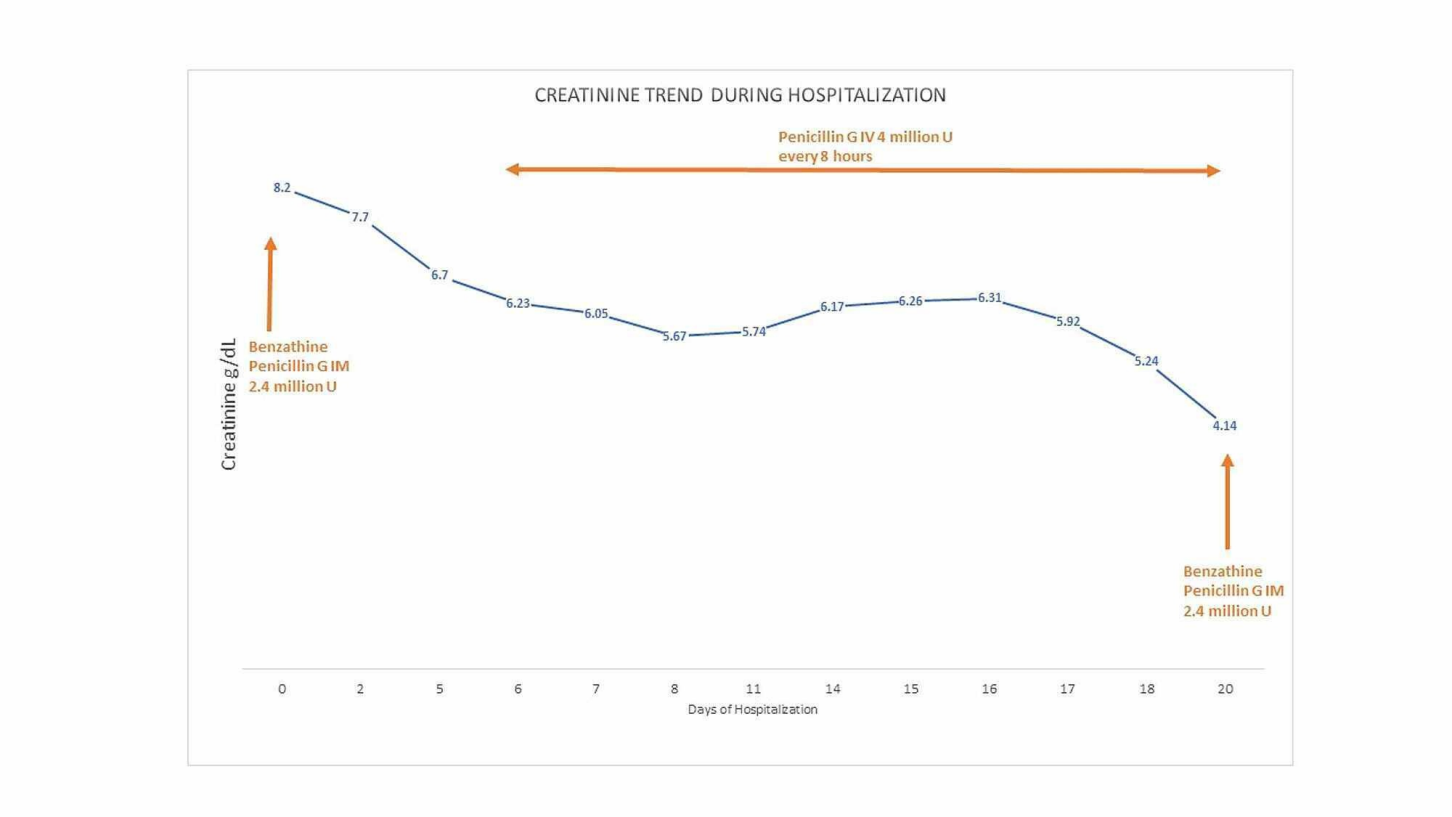
Trend of creatinine during hospitalization.

**Figure 2:**
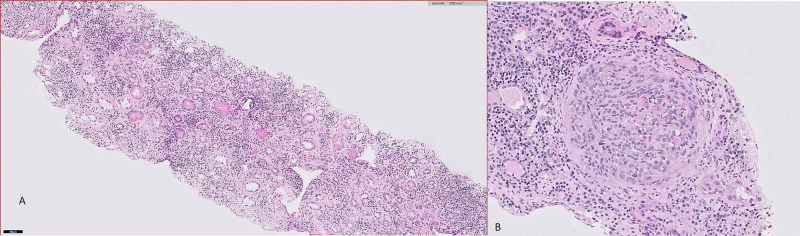
Hematoxylin and eosin staining of renal biopsy under light microscopy. (A) Dense inflammation with fibrous and cellular crescents; (B) highly magnified cellular crescents.

**Figure 3:**
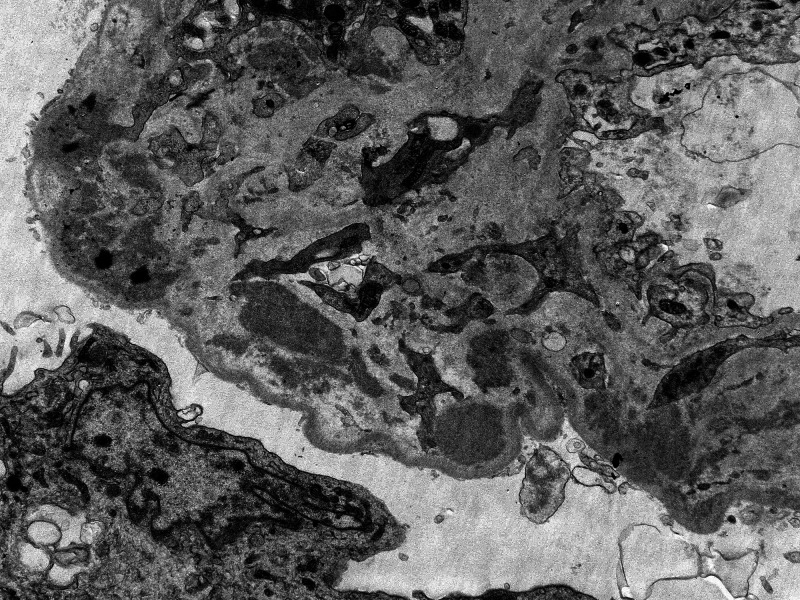
Electron microscopic image showing dense deposits in the mesangium.

A 40-year-old male with a past medical history of HIV for 10 years, currently on Triumeq (abacavir-dolutegravir-lamivudine), chronic hepatitis B virus (HBV) for two years, and chronic kidney disease stage 2 presented to a virology clinic after a gap of eight months. The patient reported severe fatigue, anorexia, weight loss, arthralgias, intermittent chills, and rash throughout the body for the last one month. The patient reported non-compliance with HIV medications, unprotected sexual intercourse, and cocaine use in the last two months. Physical examination showed diffuse scaly round macules involving the trunk, arms, legs, palms, and soles. Labs showed CD4 count 176 cells/µL (range: 503-1,144 cells/µL), HIV viral load 342,963 particles/mL, hepatitis B viral load 158 IU/mL, blood urea nitrogen 57 mg/dL (range: 7-23 mg/dL), and creatinine 8.20 mg/dL (baseline creatinine 1.47 mg/dL; normal range: 0.5-1.30 mg/dL). Urinalysis showed large blood (normal: negative), 300 mg/dL proteins (normal: negative), >50 red blood cells (RBCs)/HPF (normal range: 0-3/HPF) and 11-20 white blood cells (WBCs)/HPF (normal range: 0-4/HPF), erythrocyte sedimentation rate > 145 mm/hour (range: 0-10 mm/hour), high sensitivity C-reactive protein 41.2 mg/L (range: <5 mg/L), positive *T. pallidum* antibody screen, and positive rapid plasma reagin (RPR) with RPR titers 1:1,024 (range: <1:1 titers). The patient received treatment with benzathine penicillin 2.4 million units intramuscularly, and HIV medication Triumeq was resumed. The patient was admitted to the medical floor for a workup of acute kidney injury. Ultrasound of the kidney showed enlarged bilateral kidneys with the right kidney measuring 15.5 × 7 × 6.8 cm and the left kidney measuring 15.3 × 7.2 × 6.2 cm, suggestive of renal parenchymal disease. Creatinine started to go down slowly during the course of hospitalization (Figure 1). Further workup showed antinuclear antibody (ANA) titers of 1:320 (reference: <1:80), anti-double-stranded (anti-ds) DNA antibody 82 IU/mL (reference: <29 IU/mL), anti-smith antibody negative, anti-glomerular basement membrane antibody negative, unremarkable serum electrophoresis, glomerular proteinuria on urine electrophoresis, P-antineutrophil cytoplasmic antibodies (P-ANCA) and C-antineutrophil cytoplasmic antibodies (C-ANCA) negative, C3 complement 128 mg/dL (reference: 81-157 mg/dL), C4 complement 11 mg/dL (reference: 13-39 mg/dL), and protein/creatinine ratio in urine 4.8 (reference: 0-0.2). The patient also developed partial loss of left eye vision, and eye examination raised suspicion for neurosyphilis with retinal involvement. Cerebrospinal fluid analysis showed protein 62 mg/dL (reference: 15-45 mg/dL), glucose 60 mg/dL (reference: 40-70 mg/dL), WBCs 7 cells/µL (reference: 0-5 cells/µL), absent RBCs, and VDRL titers of 1:1 (reference: absent titers). The patient was treated with intravenous penicillin G four million units every eight hours for 14 days. The diffuse macular rash resolved completely. The biopsy of the kidney was done which on light microscopy showed fibrous and cellular crescents in most glomeruli with moderate-to-severe interstitial fibrosis and tubular atrophy (Figure 2). Electron microscopy showed dense deposits predominantly in the mesangium with scattered deposits in subepithelial areas (Figure 3). Immunofluorescence study also showed diffuse staining for c3 in the mesangium and segmentally in the basement membrane. The diagnostic impression was c3-dominant crescentic glomerulonephritis. The patient reported improvement in symptoms with a resolution of skin rash with penicillin therapy. The patient was discharged after getting another shot of benzathine penicillin G 2.4 million units intramuscularly. The kidney function continued to improve gradually, and in two weeks post-discharge follow-up, creatinine improved to 3.56 mg/dL.

## Discussion

In our patient, multiple risk factors were present that predisposed him to glomerulopathies. HIV, HBV, syphilis, cocaine use, and positive ANA and anti-ds-DNA antibodies can predispose patients to acute kidney injury with glomerulopathies. HIV infection can present with a wide spectrum of kidney manifestations such as HIV-associated focal segmental glomerulosclerosis, medication-induced nephrotoxicity, HIV-associated nephropathy, and HIV-associated immune complex kidney disease [7]. The histological patterns associated with HIV infections include mesangial proliferative glomerulonephritis, post-infectious glomerulonephritis, lupus-like glomerulonephritis, membranoproliferative glomerulonephritis, and HIV- associated IgA nephropathy [7]. Although antiretroviral medication was resumed at the same time as penicillin, the rapid onset of the improvement in kidney function, the presence of c3- dominant crescentic glomerulonephritis, and long history of HIV infection with multiple prior episodes of non-compliance without kidney involvement make HIV a less likely culprit of glomerulonephritis.

Hepatitis B infection has also shown kidney involvement with the most common histological patterns including membranous nephropathy, membranoproliferative glomerulonephritis, and polyarteritis nodosa due to deposition of hepatitis B surface antigen and E antigen in the glomeruli and circulating immune complex deposition in the vessel wall [8]. In our patient, history of hepatitis B for more than two years, low viral load of hepatitis B, normal aspartate transaminase and alanine aminotransferase, and the absence of hepatitis B-associated histological findings on kidney biopsy makes hepatitis B unlikely to be the cause of rapidly proliferative glomerulonephritis.

In our patient, positive ANA antibodies and anti-double-stranded DNA raised the suspicion for lupus nephritis. Lupus nephritis is characterized by the presence of immune complexes within glomeruli. It is divided into six classes according to the International Society of Nephrology/Renal Pathology based on the extent and location of immune-complex deposition [9]. The absence of immune-complex deposition, presence of c3-dominant glomerulonephritis, normal serum complements c3, and borderline low c4 level and improvement in kidney function without steroids made the diagnosis of lupus nephritis unlikely.

In recent times, the adulteration of cocaine with anthelmintic levamisole has contributed to pauci-immune focal and crescentic glomerulonephritis in cocaine users with almost all patients positive for P-ANCA and almost half of the patients positive for C-ANCA [10]. In our patient, both P-ANCA and C-ANCA antibodies were negative and biopsy showed c3-dominant glomerulonephritis, which makes levamisole-altered cocaine unlikely as the cause of glomerulonephritis.

Syphilis has a well-known association with kidney disease that typically presents with nephrotic-range proteinuria. The most common histological finding is membranous nephropathy. Other less common histological findings include minimal change disease, membranoproliferative glomerulonephritis, and diffuse proliferative glomerulonephritis [4]. The nephrotic syndrome associated with syphilis typically responds well to treatment with penicillin [11]. Nandikanti et al. reported a case of rapidly proliferative glomerulonephritis secondary to syphilis infection in an HIV patient with positive RPR and *Treponema* antibodies and renal biopsy findings of crescentic glomerulonephritis with only c3 deposition that improved after three weeks of treatment with weekly penicillin therapy [5]. In our patient, the concurrent presence of skin manifestations of secondary syphilis and acute kidney injury with positive urine sediment, biopsy finding of crescentic glomerulonephritis with only c3 deposits, and improvement in kidney function secondary to penicillin therapy strongly suggested that syphilis was responsible for rapidly proliferative glomerulonephritis.

## Conclusions

This case report underscores the importance of keeping syphilis high in the differentials of rapidly worsening kidney function with proteinuria and hematuria in at-risk populations. Syphilis-induced rapidly proliferative glomerulonephritis is a highly aggressive disease that can cause permanent kidney injury if not diagnosed and treated in a timely manner.
